# Erectile Dysfunction Is the Main Correlate of Depression in Men with Chronic Spinal Cord Injury

**DOI:** 10.3390/jcm10102090

**Published:** 2021-05-13

**Authors:** Arcangelo Barbonetti, Settimio D’Andrea, Chiara Castellini, Maria Totaro, Mario Muselli, Francesca Cavallo, Giorgio Felzani, Stefano Necozione, Sandro Francavilla

**Affiliations:** 1 Andrology Unit, Department of Life, Health and Environmental Sciences, University of L’Aquila, 67100 L’Aquila, Italy; dandrea.settimio@alice.it (S.D.); chiara.castellini@univaq.it (C.C.); maria.totaro@outlook.com (M.T.); sandro.francavilla@univaq.it (S.F.); 2 Epidemiology Division, Department of Life, Health and Environmental Sciences, University of L’Aquila, 67100 L’Aquila, Italy; mario.muselli@graduate.univaq.it (M.M.); stefano.necozione@univaq.it (S.N.); 3 Spinal Unit, San Raffaele Institute of Sulmona, 67039 Sulmona, Italy; francycavallo@hotmail.it (F.C.); giorgio.felzani@sanraffaele.it (G.F.)

**Keywords:** impotence, mood disorders, paraplegia, psychological health, quadriplegia, sexual dysfunction

## Abstract

Depression is the most prevalent psychological issue after a spinal cord injury (SCI) and is associated with noticeable disability, mortality and health expenditure. As SCI mainly occurs in sexually active men at a young age, and can lead to them suffering from an organic neurogenic erectile dysfunction (ED), we supposed that ED could be a major correlate of depressive status in men with SCI. As documented by a Beck Depression Inventory-II (BDI-II) score ≥14, depression was reported in 17 out of 57 men with a chronic SCI (29.8%). They exhibited a significantly higher prevalence of ED and a more severe bowel and bladder dysfunction when compared to the group without depression. At the multiple logistic regression analysis, depression showed a significant independent association with ED (OR = 19.0, 95% CI: 3.1, 203.3; *p* = 0.004) and, to a lesser extent, with a severe impairment of bowel and bladder function (OR = 0.84; 95% CI: 0.72, 0.94; *p* = 0.01). Depression was observed in 43.7% of men with ED and only in 12.0% of those without ED (*p* = 0.002). In conclusion, healthcare providers should give the right level of importance to the management of ED in men with SCI, as this represents a major independent correlate of depression, which, in turn, might hinder physical rehabilitation and exacerbate physical health issues related to SCI.

## 1. Introduction

In studies using diagnostic interviews, the prevalence of major depression in patients with SCI ranged from 9.8% to 37.5% [1]. Similar prevalence rates have been reported in both rehabilitative settings for SCI (20–43%) and community-dwelling contexts (11–60%) when screening tools for depression were used [2]. Nevertheless, in the US population, the 1-year prevalence of major depression was 6.7% [3], therefore resizing depression as a major concern in people with SCI.

Left untreated, depressive symptoms may become chronic and negatively impact the health-related quality-of-life for people with SCI, leading to secondary health disorders with recurrent hospitalizations [4], poorer functional gains [5], and reduced life expectancy [6]. Major depression predicts all-cause mortality after a SCI [7]; meanwhile, the suicide rate among this population is believed to be up to five times higher than expected in the general population in the US, Europe, and Australia [8,9]. The high prevalence and clinical weight of depression promoted research in the field of SCI, focusing on risk and protective factors for this relevant condition. Alcohol and drug misuse were significantly predictive for severe SCI-related depression [10]. A large, systematic review of observational data from 3172 adults with SCI identified relevant psychosocial correlates of post-injury depression [11]. While personal variables, such as affective feelings and SCI-specific thoughts, had the strongest relationships with depression, the acceptance of disability, life satisfaction, participation in the community and the environmental support displayed medium to strong associations [11]. These findings suggested the need to undertake early interventions and prevention approaches focusing on the evaluation and management of risk factors for psychosocial issues to limit the impact of SCI-related depression [12].

Attention has, to date, focused on psychosocial, demographic, and neurologic issues as putative major correlates of depressive symptoms in patients with SCI [5,10,11,12], largely neglecting the role of sexual dysfunction. As young men generally get involved in riskier activities than women, they are more prone to accidents, and consequently to traumatic SCI. Accordingly, 80% of SCI occurs in men at an average age that has increased from 29 years during the 1970s to 43 since 2015 [13]. Thus, SCI mainly occurs in sexually active young men for whom sexual function represents a major determinant of their quality of life, interpersonal relationships [14], psychological wellbeing [15], and life satisfaction [13,16]. Erectile dysfunction (ED), defined as “the inability to achieve and/or maintain penile erection sufficient for satisfactory sexual performance” [17], represents the main determinant of both psychological distress [15] and life dissatisfaction [16] in men with SCI. Androgen deficiency and hypovitaminosis D are regarded as further putative determinants of depression in the general population [18,19,20] and in SCI individuals [21].

In this context, the weight of ED among the putative determinants of depression in men with SCI remains to be clarified. Due to the high prevalence of neurologic ED following SCI [15,16], we surmise that ED could represent a key independent correlate of depressive symptoms in this population.

## 2. Materials and Methods

Fifty-seven men, aged 47.0 ± 17.4 years, admitted to a rehabilitation program at the San Raffaele Institute of Sulmona (Italy) because of traumatic SCI, were included in the study. Each patient gave informed consent for diagnostic evaluations. All enrolled men had a neurologically stable SCI lasting more than 1 year. None of them was under replacement therapy with testosterone or suffered from cognitive or communication disorders that could compromise the validity of their responses in the questionnaires.

Patients underwent clinical and neurologic evaluations by the same trained physician. In keeping with the International Standards for Neurological Classification of SCI from the American Spinal Injury Association (ASIA) and ASIA Impairment Scale (AIS) [22,23], patients with a complete loss of both motor and sensory function in the lowest sacral segment were categorized as grade A, whereas those with an incomplete SCI were categorized as grades B to D. Grade B: motor complete lesion with preservation of some sensation below the SCI level; grade C: preservation of both some sensation and motor function, with 50% of the muscles below the SCI level unable to move against gravity; grade D: ability of more than 50% of the muscles below the SCI level to move against gravity. Functional independence degree in activities of daily living (ADL) was assessed by the Spinal Cord Independence Measure (SCIM). This is a 19-item tool measuring the functional independence degree in performing ADL [24,25]. The SCIM weighs each function separately, giving a total score ranging from 0 (totally dependent) to 100 (totally independent). Bladder and bowel dysfunction are explored by the 6th and 7th item of the SCIM, respectively, providing a bowel/bladder SCIM sub-score.

Leisure time physical activity (LTPA) was assessed in min/week with the LTPA Questionnaire for people with SCI (LTPAQ-SCI) [26], as previously described [27,28,29]. An intensity classification chart allowed for discrimination between mild, moderate, and heavy intensity LTPA, according to the perceived effort. For each intensity degree, patients reported the number of days, over the last 7 days, which they performed LTPA at each intensity. Next, they reported the min/day they spent performing LTPA at that intensity. Finally, the total number of min/weeks of activity at each intensity was calculated as follows: number of days of activity × number of min of activity. Only the total LTPAQ-SCI score was used for analyses, as this was significantly correlated with mild, moderate, and heavy LTPAQ-SCI sub-scores [30].

According to the recommendations from the National Institute on Disability and Rehabilitation Research [31], the presence and intensity of pain was assessed by the Numerical Rating Scale (NRS). Patients rated their pain on a scale from 0 (no pain) to 10 (the worst possible pain).

Upon admission, the presence and severity of significant medical comorbidity was scored using a web-based calculator (http://www.mdcalc.com/charlson-comorbidity-index-cci (accessed on 12 May 2021)) of the age-adjusted Charlson Comorbidity Index (CCI) [32]. The CCI weights medical diagnoses for severity according to the patient’s age and provides an overall index of comorbidity. However, no patient had acute or chronic coexisting diseases which could hinder the rehabilitation program.

Body weight was measured with a professional mechanical chair scale Mod. DM2 (Wunder SA BI Srl, Monza, Italia). Height was determined with an elastic tape, segmentally measuring the distances between heel and knee, knee and hip, hip and head. Body mass index (BMI) was calculated in kg/m^2^.

Erectile dysfunction was assessed using an abridged, 5-item version of the International Index of Erectile Function (IIEF)-5 [33]. The first four items focused on erectile function while the fifth item focused on intercourse satisfaction. IIEF-5 results were able to identify the presence or absence of ED according to the National Institute of Health’s definition [17]. For each five items, a score from 0 to 5 was provided, to discriminate patients with (score ≤21) and without (score from 22 to 25) ED.

The presence of depressive symptoms and their severity were assessed by the interviewer-assisted self-report Beck Depression Inventory-II (BDI-II) [34] which was administered at admission by the same psychologist (F.C.). The BDI-II is a 21-item screening tool for depressive symptoms [19,35], largely used for people with SCI [21,36,37]. Previously published cut-off points identified patients with “no depression” (score <14) and “mild to severe depression” (≥14) [20,34].

A fasting, morning venous blood sample was obtained from each patient between 8.00 and 9.00 a.m. Total testosterone levels were measured with a chemiluminescence immunoassay, using a kit from Ortho Clinical Diagnostics (Johnson & Johnson, New Brunswick, NJ, USA). The lower limit of detection for testosterone measurement was 0.03 nmol/L and the within- and between-assay coefficients of variation (CV) were 2.5% and 4.9%, respectively. Sex hormone binding globulin (SHBG) was quantified by a chemiluminescence immunoassay, using a kit from Medical Systems (Genova, Italy). Albumin was measured by spectrophotometry using a colorimetric assay kit from Roche Diagnostics (Monza, Italy). The levels of calculated free testosterone were derived from total testosterone, SHBG, and albumin as previously described [38], using a web-based calculator (http://www.issam.ch/freetesto.htm (accessed on 12 May 2021)). Serum 25-hydroxy vitamin D (25(OH)D) levels were quantified using a chemiluminescent immunoassay (LIAISON; DiaSorin, Saluggia, Italy) with an intra- and inter-assay CV of 4.5% and 8.5%, respectively. All the other biochemical/hematologic parameters were measured using standard methods and commercial kits (Instrumentation Laboratory Co., Bedford, MA, USA).

Statistical analysis was carried out using the R statistical software (version 3.5.0, The R Foundation for Statistical Computing, Vienna, Austria). After ascertaining the non-normal distribution of data with the Shapiro–Wilk test, the Wilcoxon rank-sum test was used to evaluate differences in continuous variables between participants dichotomized as depressed (BDI-II score ≥14) or non-depressed (BDI-II score <14). Proportional differences were assessed by the chi-square test or the Fisher’s exact test, as appropriate. Multiple logistic regression analyses with odds ratios (ORs) and 95% confidence intervals (CIs) were performed to identify independent associations with depression.

## 3. Results

Depression was reported in 29.8% (17 out of 57 men) of the study population. When men were categorized by depression status, according to BDI-II score (Table 1), the depressed group exhibited significantly lower levels of both total and calculated free testosterone as well as vitamin D, a higher prevalence of ED, a more severe bowel and bladder dysfunction, and were engaged in a poorer, albeit not significantly different, LTPA when compared to non-depressed men. Specific SCI-related variables, such as level and completeness of the injury and the impairment degree in the global functional independence, were not significantly different between the two groups, and this was also the case for the demographic variables, including education and marital/partner status.

At the multiple logistic regression analysis (Table 2) which included variables which were significantly different between depressed and non-depressed men (Table 1), depression exhibited a significant independent association with ED and, to a lesser extent, with a more severe impairment of bowel and bladder function. No significant associations were found with testosterone or vitamin D levels (Table 2).

**Figure 1:**
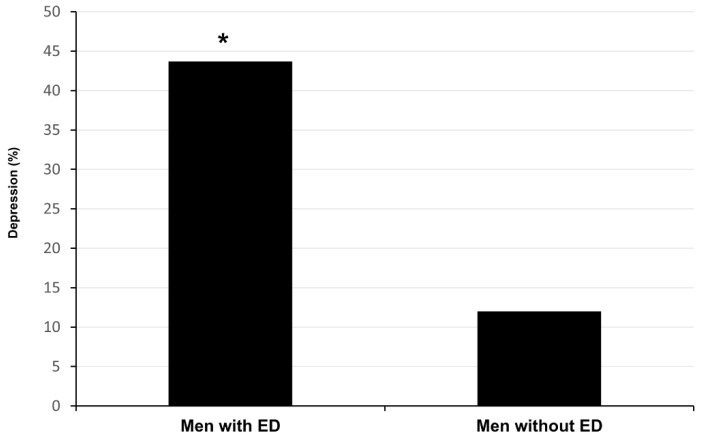
Percentage of spinal cord injured, depressed men with and without erectile dysfunction (ED). * *p* = 0.002.

As shown in Figure 1, depression was reported by 14 out of the 32 (43.7%) men with ED and only by 3 out of the 25 men (12.0%) without ED (*p* = 0.002).

## 4. Discussion

It is well known that psychological disorders are documented in able-bodied men who sought medical care for ED [39,40], showing a 2.4-fold increased risk of depression [41]. However, whether psychological distress represents an independent risk factor for ED rather than a consequence or a coexisting condition of ED is not easily established [41]. Men with SCI are affected by an organic neurogenic ED relied on the level and completeness of neurologic lesion [15]: this would explain the lack of a significant association between ED prevalence and age in this population [15]. In this light, SCI could represent an interesting clinical model to explore a possible causative link between ED and depression. Here, we showed that ED was significantly more prevalent in men with SCI reporting depressive symptoms compared to those without depression. In the multiple regression analysis, ED was the most relevant independent correlate of depressive symptoms.

Men with a traumatic SCI suffer from a sudden, violent and, in most cases, irreversible form of tragic nonlethal disease. They need to manage various problems, which will definitively affect their whole lives. Physical restrictions and the loss of functional independence are expected to represent relevant determinants of depression [42]. Therefore, it is expected that men with a more severe locomotor disability (that is tetraplegia) should exhibit a higher risk of depression compared to men with paraplegia. On the contrary, in our study of men with chronic SCI, neurological characteristics, including the level and completeness of the lesion and the physical, functional independence, were not significantly different between depressed and not depressed men, as previously reported [7,10,37,43,44,45,46]. This is in line with our previous study, in which men with a cervical lesion, despite lower functional independence, exhibited significantly lower psychological distress than those with thoraco-lumbar lesions [15]. Our findings also agree with previous reports showing that disability degree did not affect or predict perceived quality of life in people with SCI [47,48,49].

Indeed, paraplegic status was associated with a significantly higher prevalence of ED compared to tetraplegia [15]. ED, which is more prevalent and severe in thoraco-lumbar SCI than cervical lesions, might have a pivotal effect in favoring depressive symptoms, as well as life dissatisfaction, despite higher functional independence in men with a thoraco-lumbar lesion.

Besides ED, bowel/bladder dysfunction was more severe in men with depressive symptoms. While bowel and bladder dysfunction represent a relevant concern of active patients with SCI [50], potentially contributing to their psychological distress, the association of physical functional independence with depression is unclear. In a large survey of 849 patients with chronic SCI (76.0% males), the rate of a probable major depression disorder, assessed by The Patient Health Questionnaire–9 (PHQ-9), did not differ according to injury level (paraplegia vs. tetraplegia), injury severity or functional independence measure (FIM) discharge scores, which included sphincter control [1]. On the contrary, fecal impaction, a main trigger of autonomic dysreflexia, resulting from SCI-related neurogenic bowel dysfunction, was linked to depression [51] as assessed by the BDI score and was closely related to the patient’s ability to perform self-catheterization [43]. The causal link between depression and neurogenic bowel and/or bladder dysfunction outcomes is not yet elucidated.

The degree of bowel/bladder dysfunction in our survey maintained a significant independent association with depression at the multiple logistic regression analysis, although to a much lower extent than ED, thus resizing its potential relevance as a risk factor for depressive symptoms in men with SCI. In a previous study, we demonstrated that the degree of bowel/bladder independence was only significantly correlated with the degree of life satisfaction, among the SCI-related variables; this association was lost at the multiple logistic regression model adjusted for the sexual satisfaction score of the Lisat-9 questionnaire [16]. We surmised that life satisfaction was not directly affected by the neurogenic impairment of bowel and/or bladder function, which adversely impacted sexual well-being. Indeed, anal sphincter dysfunction causing recurrent accidental bowel leakage during sexual activity may result in an obvious embarrassment in life relationships. Similarly, sexual intercourse may be hindered or impeded by the need for an indwelling catheter because of severe neurogenic bladder dysfunction. Here, we extended this conclusion, demonstrating that neurogenic bowel/bladder dysfunction also has an independent effect on symptoms of depression.

Interestingly, total and calculated free testosterone levels were significantly lower in spinal cord injured men with depression when compared to the non-depressed group, but this association was no longer present when the logistic regression model was adjusted for ED. The contribution of erectile function, irrespective of the androgen status, is not surprising. ED represents a major attribute of androgen deficiency in the general population [52], but not in men with SCI [30] who, as mentioned, suffer from a purely organic neurogenic ED depending on the level and completeness of SCI [15]. ED would accordingly contribute to depression independently of testosterone levels.

We previously demonstrated that vitamin D levels were inversely associated with the risk of depression in people with SCI [21]. In the present study, depressed men with SCI exhibited lower vitamin D levels compared to men who were not depressed. However, this association was lost after adjusting for ED and sphincter dysfunction. Depression itself may contribute to lower vitamin D levels by reducing outdoor physical activity and, consequently, sunlight exposure. Hence, other than a direct causal role of hypovitaminosis D in depression, both conditions could represent markers of poor health status, without a direct interaction.

This study has some limitations that must be considered. The cross-sectional study design could not establish the cause-effect relationship between ED and depression. Moreover, the IIEF-5 was not validated for SCI and, therefore, in the present population it does not discriminate between reflexogenic and psychogenic erection. However, IIEF-5 has already been used for men with SCI [53,54]. Finally, the sample size of our series was quite small, especially in the depressed group, but was sufficient to demonstrate probable significant independent associations due to the high prevalence of the investigated disorders among men with chronic SCI.

In conclusion, ED and, to a lesser extent, bowel/bladder dysfunction in men with SCI, showed a significant and independent association with depression, pointing to a possible causal role of ED on depressive symptoms in this population and confirming that, in sexually active men, the restoration of sexual life could rate as a high priority. Longitudinal studies should explore whether the appropriate management of ED and sphincter dysfunction might prevent depression while fostering life satisfaction in men with SCI.

## Figures and Tables

**Table 1:** Characteristics of the study population categorized by depression status at the Beck Depression Inventory-II.

Characteristics	Beck Depression Inventory-II Score		*p* Value
	<14 (Not Depressed) N = 40	≥14 (Depressed) N = 17	
**Demographic and lifestyle variables**			
Age (years)	41.5 (21.0–81.0)	52.0 (20.0–76.0)	0.5
Education—*n* (%)			
Primary school	11 (27.5)	7 (41.2)	0.6
High school	24 (60.0)	9 (53.0)	
University	5 (12.5)	1 (5.8)	
Marital/partner status—*n* (%)			
Single	18 (45.0)	6 (35.3)	0.7
Married	16 (40.0)	7 (41.3)	
Divorced/separeted	4 (10.0)	2 (11.7)	
Widowed	2 (5.0)	2 (11.7)	
LTPA (min/week)	560 (35–1225)	245 (35–1015)	0.08
**Clinical and injury-related characteristics**			
BMI (Kg/m^2^)	24.6 (15.2–37.2)	25.4 (12.3–32.7)	0.5
Charlson Comorbidity Index score	2 (0–8)	3 (0–7)	0.2
BDI-II score	6.5 (1–13)	14 (14–23)	<0.0001
Psychotropic drugs—*n* (%)	12 (30.0)	6 (35.3)	0.9
Duration of injury (years)	11.2 (1.5–37.0)	7.0 (1.1–24.0)	0.1
Lesion motor completeness—*n* (%)			
Complete (AIS A+B)	25 (62.5)	14 (82.3)	0.2
Incomplete (AIS C+D)	15 (37.5)	3 (17.7)	
Level of the lesion—*n* (%)			
Cervical Spine	19 (47.5)	11 (64.7)	0.3
Thoracic-lumbar Spine	21 (52.5)	6 (35.3)	
SCIM score	38.0 (11.0–56.0)	35.0 (10.0–69.0)	0.4
Bowel/bladder SCIM sub-score *	22.0 (0.0–25.0)	9.0 (0.0–20.0)	0.001
Pain Intensity (NRS score)	3.0 (0.0–10.0)	3.0 (0.0–8.0)	0.9
Erectile dysfunction—*n* (%)	18 (45.0)	14 (82.3)	0.02
**Blood biometric measures**			
Total testosterone (ng/dL)	404.5 (111.0–713.0)	268.0 (25.0–694.0)	0.03
Calculated free testosterone (pg/mL)	118.1 (27.8–241.1)	65.0 (2.9–162.9)	0.02
Vitamin D (ng/mL)	16.3 (4.1–34.4)	11.1 (4.4–22.6)	0.02
**Season of evaluation—*n* (%)**			
Autumn/winter	24 (60.0)	7 (41.2)	0.3
Spring/summer	16 (40.0)	10 (58.8)	

Data were expressed as median (minimum-maximum) for continuous parameters and as number (%) when categorical. * The bowel/bladder SCIM sub-score included the 6th and 7th items of the SCIM. Abbreviations: AIS, American spinal injury association (ASIA) Impairment Scale, BDI-II, Beck Depression Inventory-II, BMI, body mass index, LTPA, leisure time physical activity, NRS, numeric rating score, SCIM Spinal Cord Independence Measure.

**Table 2:** Multiple logistic regression analysis of the independent correlates of depression in men with chronic spinal cord injury.

	Depression (BDI-II Score ≥14)	
	OR (95% CI)	*p* Value
Erectile dysfunction (IIEF-5 score ≤21)	19.0 (3.1; 203.3)	0.004
Bowel/bladder SCIM sub-score	0.84 (0.72; 0.94)	0.01
Calculated free testosterone (pg/mL)	0.98 (0.96; 1.0)	0.07
Vitamin D (ng/mL)	0.96 (0.82; 1.1)	0.6

To convert the values for calculated free testosterone to pmol/L, multiply by 3.467. Abbreviations: BDI-II, Beck Depression Inventory-II; CI, confidence intervals; IIEF-5, international index of erectile function 5; OR, odds ratio.

## Data Availability

All data are contained within the article. No additional information is available for data sharing.
